# An all optical approach for comprehensive in-operando analysis of radiative and nonradiative recombination processes in GaAs double heterostructures

**DOI:** 10.1038/s41377-022-00833-5

**Published:** 2022-05-13

**Authors:** Fan Zhang, Jose F. Castaneda, Timothy H. Gfroerer, Daniel Friedman, Yong-Hang Zhang, Mark W. Wanlass, Yong Zhang

**Affiliations:** 1 Department of Electrical and Computer Engineering, The University of North Carolina at Charlotte, Charlotte, NC 28223 USA; 2 Songshan Lake Materials Laboratory, Dongguan, Guangdong 523808 China; 3 Department of Physics, Davidson College, Davidson, NC 28035 USA; 4 National Renewable Energy Laboratory, Golden, CO 80401 USA; 5 School of Electrical, Computer and Energy Engineering, Arizona State University, Tempe, AZ 85287 USA; 6 Wanlass Consulting, Norwood, CO 81423 USA

**Keywords:** Electronics, photonics and device physics, Solar energy and photovoltaic technology

## Abstract

We demonstrate an *all optical* approach that can surprisingly offer the possibility of yielding much more information than one would expect, pertinent to the carrier recombination dynamics via both radiative and nonradiative processes when only one dominant deep defect level is present in a semiconductor material. By applying a band-defect state coupling model that explicitly treats the inter-band radiative recombination and Shockley–Read–Hall (SRH) recombination via the deep defect states on an equal footing for any defect center occupation fraction, and analyzing photoluminescence (PL) as a function of excitation density over a wide range of the excitation density (e.g., 5–6 orders in magnitude), in conjunction with Raman measurements of the LO-phonon plasmon (LOPP) coupled mode, nearly all of the key parameters relevant to the recombination processes can be obtained. They include internal quantum efficiency (IQE), minority and majority carrier density, inter-band radiative recombination rate (*W*_r_), minority carrier nonradiative recombination rate (*W*_nr_), defect center occupation fraction (*f*), defect center density (*N*_t_), and minority and majority carrier capture cross-sections (*σ*_t_ and *σ*_tM_). While some of this information is thought to be obtainable optically, such as IQE and the *W*_r_/*W*_nr_ ratio, most of the other parameters are generally considered to be attainable only through electrical techniques, such as current-voltage (I-V) characteristics and deep level transient spectroscopy (DLTS). Following a procedure developed herein, this approach has been successfully applied to three GaAs double-heterostructures that exhibit two distinctly different nonradiative recombination characteristics. The method greatly enhances the usefulness of the simple PL technique to an unprecedented level, facilitating comprehensive material and device characterization without the need for any device processing.

## Abstract (web summary)

A measurement of PL intensity vs. excitation density, with the help of Raman, is shown capable of determine nearly all pertinent parameters of radiative and nonradiative recombination in a semiconductor.

## Graphical abstract

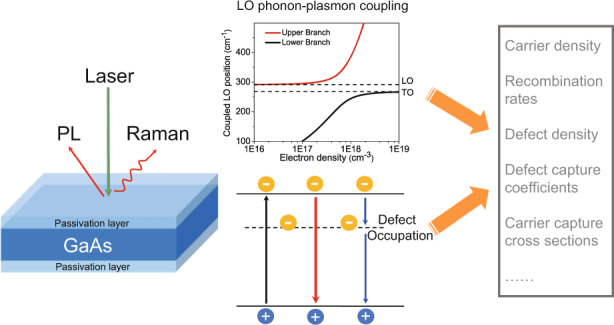

## Introduction

To determine the carrier densities in a semiconductor, electrical characterization techniques, for instance Hall measurements, are typically used through Ohm’s law *J* = *qnμE*^1^, where *J* is the current density, *q* the carrier charge, *n* the carrier density, *μ* the mobility, and *E* the electrical field. To determine the characteristics of nonradiative recombination centers, another electrical technique, deep level transient spectroscopy (DLTS)^2^, is often used. For these electrical measurements, contacts and thus device processing are required, which might not be practical for many situations. Electrical measurements also become impractical in certain circumstances, such as with a doped substrate, particularly under optical excitation or with external carrier injection. Photoluminescence (PL) spectroscopy, a widely used material characterization technique, is normally used for qualitative assessment of material quality, and thus, it is not considered a viable tool for obtaining quantitative information about material properties or in-operando device behavior, such as absolute carrier densities and nonradiative center capture rates^3^, although some qualitative information regarding nonradiative centers can be obtained by performing both excitation density and temperature dependent PL^4^. Recently, we have shown that by analyzing PL intensity vs. excitation density over multiple orders in excitation density, one is able to extract the PL internal quantum efficiency (IQE) vs. excitation density^5^, and by combining PL and Raman mapping, one is able to obtain the spatial distribution of the electron density near individual dislocation defects under optical excitation^6^. Here in this contribution, we take another major step forward from the previous work^5,6^, demonstrating an all optical approach combining continuous-wave (CW) PL and Raman that offers the possibility for carrying out a comprehensive analysis of radiative and nonradiative properties of a semiconductor over a wide range of excitation or carrier density, including IQE, minority and majority carrier density, inter-band radiative recombination rate and coefficient, defect center occupation fraction, defect center density, and minority and majority carrier capture cross-section or rate.

In either PL or EL (electroluminescence), IQE is defined as the ratio between the band edge radiative recombination rate and total recombination rate, which includes all possible recombination processes, and in particular, nonradiative recombination through defects. Under continuous excitation (by either incident light or injected current), the steady state distribution of electrons among different energy states will be established as a result of balancing different competing processes. For instance, the carrier density in the conduction band depends on both the radiative recombination rate and the capture rate of the defects, and the latter depends on the occupation level of the defect states, which then depends on the depletion rate of the defect states through recombination with the holes in the valance band (also known as the hole capture rate). Although the excitation rate can be determined or estimated relatively easily (e.g., the number of photons being absorbed or the electrons being injected into the circuit per unit time), and the emission intensity (i.e., the rate at which photons are emitted) can be directly measured with proper calibration, it is not straightforward to determine the *operando* carrier densities in the conduction and valance band because they usually cannot be directly measured. However, the carrier densities are often needed for quantifying physical processes and device modeling. Therefore, it is of great value to develop a method to accurately determine the carrier density in a semiconductor under excitation. Additionally, it is highly desirable in the meantime to have the ability to also obtain quantitative information regarding detrimental point defects and assess their effects under different excitation conditions using a simple optical approach.

In the literature, an ABC model has been used as an independent method to estimate the IQE and carrier density in semiconductors. It describes the recombination processes of the excited carriers with the formula *G* = *An* + *Bn*^2^ + *Cn*^3^, where *G* represents the total carrier generation rate, *n* = *p* is the free carrier density, and the three recombination terms are nonradiative (*An*), radiative (*Bn*^2^), and Auger (*Cn*^3^), with *A*, *B*, and *C* being constants^7–10^. Because 100% IQE implies a linear relation between emission intensity (*I*_PL_ ∝ *Bn*^2^) and excitation intensity (*P*), it is possible to obtain IQE by fitting the observed *I*_PL_ vs. *P* curve to a nonlinear function IQE(*P*) = *Bn*^2^/*G*^5,8,10,11^. Because of the assumed simplistic bimolecular radiative recombination rate *Bn*^2^, the ABC model can further yield the *n* vs. *P* relation if the radiative recombination coefficient *B* is known^8^. However, this model has a few shortcomings, including: (1) it assumes that the three processes are independent, (2) it assumes that *A* is a constant, and (3) it makes conflicting assumptions for the *A* and *B* terms. The independence assumption might be valid for a very low excitation level, but in that case the free electron and hole densities *n* and *p* will differ, depending on the defect state occupation level and background doping. Because the *A* term representing Shockley–Read–Hall (SRH) recombination^12,13^ is often saturable with increasing excitation level^5,14,15^, the nonradiative recombination rate will not change linearly with *n* when the excitation density varies over multiple orders in magnitude, as assumed by the ABC model. The selective capture of electrons or holes into trapped states implies *n* ≠ *p*, and thus *Bn*^2^ will not be generally valid over a wide range of excitation levels. By including the rate equation for the defect state, a three-level model (referred to as the coupling model hereafter) has been proposed to overcome the above-mentioned shortcomings and address the strong coupling between the band and defect states to describe defect-related recombination^15^, and further to obtain the IQE curves for rather complex and very different PL vs. *P* curves for various CdTe, GaAs, and hybrid perovskite samples^5^. However, the carrier density and defect state information remain elusive when analyzing the PL data alone.

Raman scattering of the longitudinal optical phonon-plasmon (LOPP) coupled mode^16,17^ has the ability to measure the free carrier density in a doped sample, particularly for electrons, and matches well with Hall and capacitance-voltage measurements^18,19^, while being more immune to the interference of other layers and requiring no additional device processing. It can also be used to characterize photo-generated carriers^20,21^. The technique has a unique ability to offer high spatial resolution, as demonstrated recently with the spatial distribution of photo-generated carrier densities near individual dislocation defects^6,22^. However, this method is only applicable over a limited range of carrier density. Nevertheless, the LOPP coupled mode Raman results may provide the only needed scaling parameter for the comprehensive analysis of the radiative and nonradiative properties of the material using PL, when a key constraint: a charge neutrality condition, is also applied.

In the present work, using GaAs as a prototype system, and by combining power-dependent PL and LOPP Raman, we are able to determine not only IQE but also carrier concentrations over the excitation power range of multiple orders, and furthermore obtain important information regarding the nonradiative centers. A characteristic feature of the excitation power dependence of IQE on *n* is shown to be a result of the interplay of defect centers with background and photo-generated carriers, and the divergence into seemly very different behaviors is explained.

## Results

Despite that GaAs is one of the most studied semiconductor materials, GaAs samples grown by different growth systems may exhibit vastly different behaviors as far as the non-radiative recombination process is concerned. Here we use three samples, WA540, 1-1138, and B2206, that show distinctly different functional dependences of the PL intensity vs. excitation density to illustrate the effectiveness and limitation of this all-optical approach and the detailed procedures to reaching the goal. They are all double heterostructures (DHs) with a thin GaAs layer sandwiched between two higher bandgap barrier layers (GaInP or AlGaAs). The first two samples were grown by MOCVD but using different machines, and the last one by MBE (see “Materials and methods” for details). The use of the DHs provides 2 benefits: (1) it suppresses the surface recombination, thus allowing for probing the bulk defects; and (2) the DH is commonly adopted in real devices.

### LO phonon-plasmon (LOPP) coupled mode

**Fig. 1:**
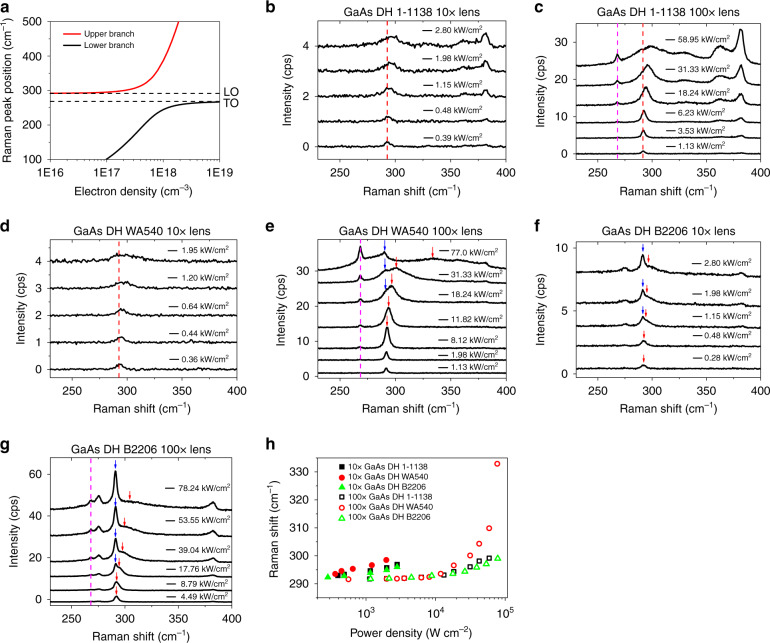
Excitation power dependent Raman data. **a** Simulated curves for electron density-dependent LOPP coupled mode frequencies in GaAs. **b**, **c** Raman spectra of 1-1138 under 10× lens and 100× lens; **d**, **e** Raman spectra of WA540 under 10 × and 100× lens; **f**, **g** Raman spectra of B2206 under 10× lens and 100× lens. Red arrows and dashed lines are coupled LO mode; purple dashed line is TO mode; blue arrows point to LO mode from depletion layer. **h** Power-density dependent LOPP coupled mode frequencies for all GaAs samples under 10× and 100× lenses

In GaAs, above-bandgap excitation generates an electron plasmon which couples with the LO phonon, producing two coupled modes, as illustrated in Fig. 1a by the two anti-crossing branches. The free carrier density *n* is linked to the plasmon frequency by Eq. 1

1 $$\omega _{{{\mathrm{p}}}} = \sqrt {\frac{{4\pi ne^2}}{{m ^* {\it{\epsilon }}_\infty }}}$$

where *ω*_p_ is the plasmon frequency, *e* is the elementary charge, m* (= 0.067 *m*_e_) is the effective mass of electrons in GaAs, and *ε*_∞_ is the high frequency dielectric constant 10.86. Dispersion curves, *ω*_±_ vs. *n*, of the coupled modes in Fig. 1a are given by Eq. 2

2 $$\omega _ \pm ^2 = \frac{1}{2}\left\{ {\omega _{{{{\mathrm{LO}}}}}^2 + \omega _{{{\mathrm{p}}}}^2 \pm \left[ {\left( {\omega _{{{{\mathrm{LO}}}}}^2 + \omega _{{{\mathrm{p}}}}^2} \right)^2 - 4\omega _{{{\mathrm{p}}}}^2\omega _{{{{\mathrm{TO}}}}}^2} \right]^{1/2}} \right\}$$

where *ω*_TO_ and *ω*_LO_ are the vibrational frequencies of the transverse and longitudinal modes of the pure optical phonons. In this work, *ω*_TO_ and *ω*_LO_ are measured to be 268.0 and 291.5 cm^−1^ from a Cr doped GaAs wafer in which almost all photo-excited carriers are depleted by nonradiative centers. These values are consistent with the literature values, 268.1 and 291.4 cm^−1,^ ^23^, within the experimental uncertainty. Upper *ω*_+_ and lower *ω*_−_ branches in Fig. 1a can be directly used to determine the electron density n from the peak position of the *ω*_+_ Raman mode. *ω*_−_ is often too weak to be measurable. Here we have neglected the damping effects because none of the GaAs samples are heavily doped and the electron instead of hole concentration is our focus^24^.

Figure 1b shows the Raman spectra of 1-1138 under the 10× lens from a power density of 0.39–2.8 kW cm^−2^. With increasing laser power density, the LO mode is blue shifted from 293.1 cm^−1^ at 0.39 kW cm^−2^ to 296.9 cm^−1^ at 2.8 kW cm^−2^, as expected for the LOPP model. In Fig. 1b, the 362 and 382 cm^−1^ bands are attributed to the InP and GaP-like LO modes of GaInP^25^, while the GaAs TO mode is nearly absent because of the Raman selection rule for (001) GaAs and weak signals. Figure 1c shows the Raman spectra of the same sample under the 100× lens where a higher numerical aperture allows for more efficient Raman signal collection. The forbidden TO mode of GaAs appears at ~268 cm^−1^ for power densities above 18 kW cm^−2^. We note that the *ω*_+_ mode blue-shift is small below 6 kW cm^−2^, but increases rapidly after that, suggesting that nonradiative defects have been saturated at higher excitation densities, leading to a quicker increase of the free electron density. Interestingly, comparison of the Raman shifts from Fig. 1b, c, as summarized in Fig. 1h, reveals that the Raman shift and the corresponding carrier density with the 100× lens are significantly lower than those with the 10× lens for the same excitation density, e.g., at 1.1 kW cm^−2^, 291.6 cm^−1^ and 3.4 × 10^15^ cm^−3^ for 100× vs. 294.6 cm^−1^ and 8.6 × 10^16^ cm^−3^ for 10×, due to the difference in the influence of carrier diffusion. The corresponding results for WA540 are given in Fig. 1d, e, respectively, under the 10× and 100× lens. Under 100×, because of the thinner top barrier layer, the Raman signal from GaInP is suppressed, which extends the power density range for observing the GaAs *ω*_+_ mode up to 77 kW cm^−2^. For the higher power region (31 and 77 kW cm^−2^), a small peak at ~291 cm^−1^ (the GaAs LO mode) appears, resulting from the surface depletion region due to the thin capping layer^26^. At lower power density, this component is convoluted with the *ω*_+_ mode. Figure 1f, g shows the results for B2206 under the 10× and 100× lens, respectively. Compared to GaAs/GaInP DHs, GaAs/AlGaAs DHs are more likely to exhibit co-existing LO and *ω*_+_ modes of GaAs due to the carrier depletion near the GaAs/AlGaAs interface^21,27^. In this sample, the LO mode at 291.2 cm^−1^ from the depleted region overwhelms the possible LO-plasmon coupled mode at low excitation density. Only at high powers, e.g., above 17 kW cm^−2^ under 100×, the coupled mode appears and becomes substantially blue shifted with increasing excitation level. We have deconvoluted the spectra to separate the two peaks. The *ω*_+_ mode peak positions obtained from Fig. 1b–g are summarized in Fig. 1h. They will be used to calculate the corresponding electron densities, which are pivotal for extracting quantitative information on radiative and nonradiative recombination from the PL data.

### Coupling model of band and defect states

**Fig. 2:**
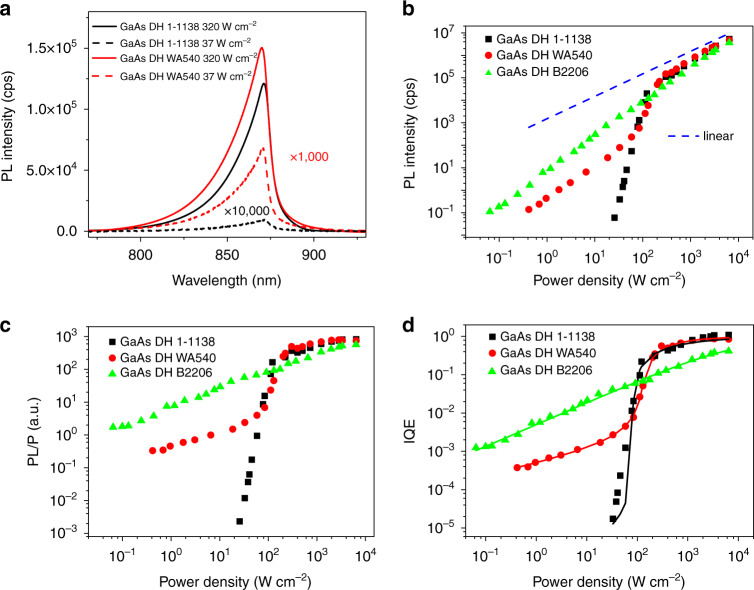
Excitation power dependent PL data (with 10× lens). **a** Typical PL spectra of 1-1138 and WA540 at two power densities; **b**–**d** PL intensity vs. power density (*P*); PL/*P* vs. *P*; and IQE vs. *P* for all three samples. Solid dots are experimental data; solid lines are fitting curves with the coupling model

Figure 2a shows selected PL spectra of 1-1138 and WA540 at 37 and 320 W cm^−2^ under the 10× lens. Although the power density differs by less than a factor of 10, the PL intensity varies by 5 and 3 orders of magnitude for 1-1138 and WA540, respectively, suggesting strong nonlinearity caused by defect filling effects^28^. More spectra for the three samples are given in Fig. S1 (Supporting Information, SI). The power dependence for all three samples under 10× is displayed in Fig. 2b. The curves are somewhat similar in the high excitation density region, but distinctly different in the low density region. The two MOCVD samples exhibit strong nonlinearity below ~200 W cm^−2^, whereas the MBE sample (B2206) shows weaker nonlinearity spread over the whole excitation range. It is interesting to note that in the log-log plot, WA540 has two regions of smaller slopes in the low and high excitation density, and a super-linear transition region between them. A somewhat similar super-linear dependence has been observed before in InGaAsP/InP DH samples^29^.

To analyze the results in Fig. 2b more quantitatively, we apply the coupling model that can better describe the competition between the inter-band radiative recombination and non-radiative recombination through defect centers under different generation levels. The data of Fig. 2b can be converted into relative external quantum efficiency (EQE), ***η***_EQE_ = *I*_PL_/*P*, where *P* is the power density, as shown in Fig. 2c. As illustrated previously (see Note 1 in SI)^5^, the data of Fig. 2c can be fit well by the function below:

3 $$\eta _{{\rm{EQE}}} = \frac{C}{2}\left( {1 - \frac{{\left( {1 + \mu } \right)\beta }}{P} + \sqrt {\left( {1 + \frac{{\left( {1 + \mu } \right)\beta }}{P}} \right)^2 - \frac{{4\beta }}{P}} } \right)$$

where *C* is a constant taking care of the light extraction efficiency and system response, *β* *=* *N*_t_*W*_t_, *μ* = *W*_r_*/c*_t_, and *P* the net incident laser power density (after correcting for reflection loss), with *N*_t_ being the total defect state density, *W*_t_ the recombination rate of a captured minority carrier at the defect state with majority carriers, *W*_r_ the inter-band radiative recombination rate of the minority carrier, *c*_t_ = *γ*_t_*N*_t_ the maximum minority carrier capture rate by the defects, and *γ*_t_ the corresponding capture coefficient. Assuming the material is p-type and considering that the majority carrier density *p* is power dependent, we introduce an empirical dependence *p* = *p*_0_(1 + *dP*^*ζ*^), with *p*_0_ being the background majority carrier density and *δp*/*p*_0_ = *dP*^*ζ*^ describing the relative number of the non-equilibrium majority carrier with *d* and *ζ* as fitting parameters. Accordingly, we have *W*_r_ = *W*_r0_(1 + *dP*^*ζ*^) and *W*_t_ = *W*_t0_(1 + *dP*^*ζ*^), where *W*_r0_ = *Bp*_0_ with *B* being radiative recombination coefficient, *W*_t0_ = *B*_t_*p*_0_ with *B*_t_ being the *B* equivalence for the defect state. Idealistic bi-molecular recombination with *n* = *p* implies *ζ* = ½, however, *ζ* ≠ ½ allows us to empirically treat non-idealistic situations, such as low excitation level or more than one majority carrier capturing state. *η*_IQE_ is related to *η*_EQE_ through *η*_IQE_ = *η*_EQE_*/C*. When *μ* ≫ 1, *η*_*IQE*_ → 1. We note that in Eq. 3 the minority carrier recombination processes are considered. If the material is n-type, the symbol *p* used above should be understood as the majority carrier *n*.

All three 10× data sets can be fit well with Eq. 3, particularly WA540 and B2206, using the fitting parameters (*μ*_0_, *β*_0_, *d*, *ζ*, *C*) given in Table 1. IQE can be obtained using *η*_IQE_ = *η*_EQE_*/C*, as shown in Fig. 2d. Under high generation rates, corresponding to the high density region in Fig. 2b (roughly above 200 W cm^−2^), the defect centers are being saturated for WA540 and 1-1138 (to be elaborated below), and IQEs approach unity at an excitation density of around 6.5 kW cm^−2^: 93% and 84% for WA540 and 1-1138, respectively. For 1-1138, the few “data points” slightly above 100% IQE represent the uncertainty of fitting (the data points were scaled by the parameter *C*). However, for B2206, IQE only reaches 43% at 6.5 kW cm^−2^. IQEs fall very quickly for WA540 and 1-1138 when *P* is below 100 W cm^−2^, particularly for 1-1138 where the fitting is not as good as for the other two samples and the fitting parameters should be considered as one plausible set, because of limited low-density data. Interestingly, B2206 is much more efficient than the other two samples below 100 W cm^−2^. These results indicate that the relative PL intensity alone at an arbitrary excitation level is not a good indicator of the quality of a material. The equivalent results of Fig. 2b–d for the 100× lens can be found in Fig. S2a–c.

**Table 1:** Fitting parameters to Eq. 3 and derived radiative and nonradiative parameters.

Parameters	WA540	1-1138	B2206
*C*	881.27	752.28	1330.89
*μ*_0_	3.64E−7	1.07E−7	8.01E−5
*β*_0_(W cm^−2^)	1.90E−2	1.24	77.42
*d*	1.35E3	4.97	62.88
*ζ*	0.33	0.58	0.58
Background doping (cm^−3^)	*p*_0_: 2.20E13	*n*_0_: 2.79E14	*n*_0_: 1.88E13
*N*_t_(cm^−3^)	1.73E17	1.31E16	1.04E19
*c*_t_(s^−1^)	9.01E10	4.24E12	6.33E8
*c*_M_(s^−1^)	4.64E7	5.96E7	1.10E11
*γ*_t_(cm^3^s^−1^)	5.21E−7	3.22E−4	6.11E−11
*B*_t_(cm^3^s^−1^)	2.68E−10	4.56E−9	1.06E−8
*σ*_t_(cm^2^)	8.68E−15	1.79E−11	3.39E−18
*σ*_tM_(cm^2^)	1.49E−17	7.58E−17	1.77E−16
*B*(cm^3^s^−1^)	1.49E−9	1.62E−9	2.70E−9

The ABC model can fit the *I*_PL_ vs. *P* data rather well for a small range of injection levels, yielding an IQE curve^8,10^. In this analysis, one can write *η*_EQE_ = *k*/(1 + *a*/√*I*_PL_ + *c*√*I*_PL_), where *k*, *a* and *c* are three fitting parameters, and *η*_IQE_ = *η*_EQE_/*k*. For comparison, we applied the ABC model to the data of the three samples in the whole power density range and obtained the corresponding IQE curves shown in Fig. S3. We find that it gives a rather poor fit in each case in the low excitation region. If one would limit the fitting to the high excitation region, the ABC model could actually fit the data fairly well, but the resulting maximum IQE values of the ABC model are still problematic: 19% (ABC) vs. 93% (coupling) for WA540 and 66% (ABC) vs. 43% (coupling) for B2206, because in reality WA540 is significantly more efficient than B2206 in the high power region.

One important aspect of the coupling model is to consider the defect level occupation level *f* that is given below:

4 $$f = \frac{P}{{2\beta }}\left( {1 + \frac{{\left( {1 + \mu } \right)\beta }}{P} - \sqrt {\left( {1 + \frac{{\left( {1 + \mu } \right)\beta }}{P}} \right)^2 - \frac{{4\beta }}{P}} } \right)$$

**Fig. 3:**
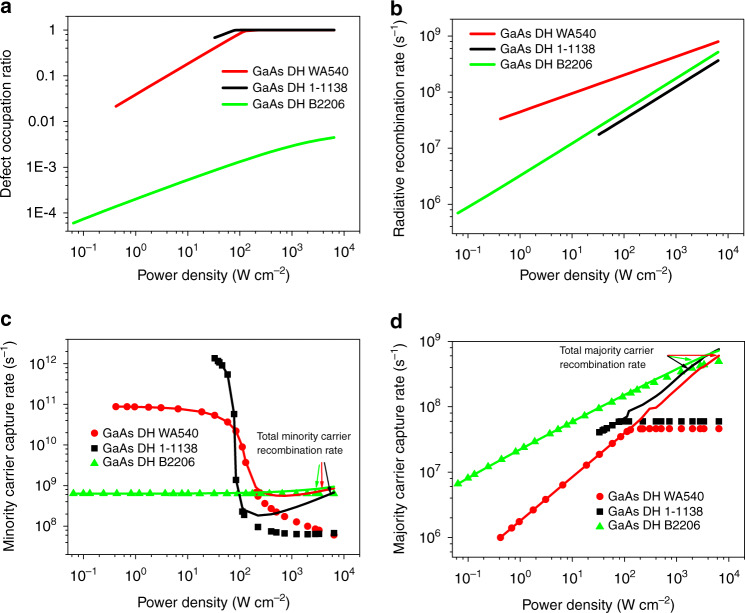
Derived transition rates for all GaAs samples under 10× lens. **a** Defect center occupation fraction, **b** Inter-band radiative recombination rate *W*_r_, **c** minority carrier defect capture rate *W*_nr_ (symbols) and total recombination rate *W* (solid lines), and **d** majority carrier defect capture rate *R*_t_ (symbols) and total recombination rate *R* (solid lines)

With all of the parameters being obtained already, we can plot *f* vs. *P*, as shown in Fig. 3a for the results of the 10× excitation. The defect filling effect can be clearly observed for the two MOCVD samples WA540 and 1-1138 as *P* reaches around 100 W cm^−2^, whereas for the MBE sample B2206, the defects are not saturated even at the highest power density, which explains its relatively low IQE at the highest power (43%). However, with PL data alone, we cannot obtain more quantitative insight regarding the defects in these three samples. More in depth analysis requires combining the PL and Raman results, and critically also applying another constraint: the charge neutrality condition.

### Combined analysis of PL and Raman results

**Fig. 4:**
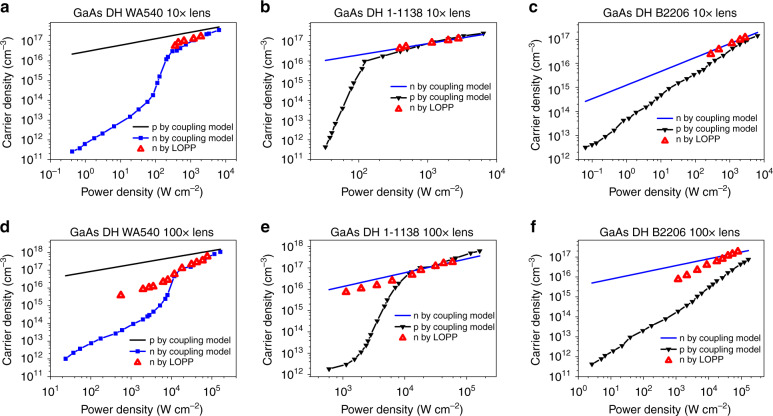
Derived photo-excited carrier densities with PL and Raman: coupling model (black for holes and blue for electrons) and LOPP (red). **a** WA540 under 10× lens; **b** 1-1138 under 10× lens; **c** B2206 under 10× lens; **d** WA540 under 100× lens; **e** 1-1138 under 100× lens; **f** B2206 under 100× lens

By applying Eq. 2, the LOPP mode Raman frequencies in Fig. 1h are used to calculate the corresponding *n* vs. *P* dependences, as shown in Fig. 4a–f for the three samples using 10× and 100× lens, respectively. Since the outward diffusion of the photo-generated carriers from the excitation site can be treated as non-radiative recombination loss in the confocal PL measurement, the results for the two lenses are expected to be rather different. Indeed, the comparison between Fig. 4a, d for WA540, 4b, e for 1-1138, 4c, f for B2206, shows that under the same power density, the electron density determined from the 10× lens is significantly higher than that from the 100× lens, indicating that the 10× lens measurement is less influenced by carrier diffusion^5^. The electron densities obtained here will be used below with the PL data to yield comprehensive information on the radiative and nonradiative recombination in the materials.

The *I*_PL_ vs. *P* curve may also be used to extract the free electron density using the ABC model by assuming that *n* = *p* and *B* is known^8,11^. However, one cannot expect the assumed bimolecular radiative recombination form to hold true over a broad range of excitation levels, particularly in the low-density region. In the coupling model, the radiative recombination rate does not take a simple bimolecular form, which makes the determination of the carrier density from the PL data alone impossible. Specifically, we may write

5 $$n = \eta _{{\rm{IQE}}}G/W_{{{\mathrm{r}}}}$$

where *n* is the minority carrier density, *η*_IQE_(*P*) has been obtained from the fitting, *G* = *P*/(*E*_ph_*t*) with *E*_ph_ being the excitation photon energy and *t* the GaAs epitaxial layer thickness (*t* ≪ carrier diffusion length). However, *W*_r_(*P*) depends on an unknown constant *W*_r0_ = *Bp*_0_ because the fitting has only given *μ*_0_ = *W*_r0_*/c*_*t*_. Nevertheless, it is significant to note that, apart from the scaling constant *W*_r0_, the coupling model can in fact determine the minority carrier density curve *n* vs. *P* over the whole excitation density range, which has not been possible previously. There are a few options to determine the scaling constant in order to determine the absolute carrier densities, for instance, (i) obtaining the background doping level independently, if *B* is also known; and (ii) scaling the electron density vs. *P* curve to the electron density derived from the LOPP Raman result at an appropriate high excitation density level. Here we adopt the second method.

If the material is p type, as for WA540, the minority carrier density *n* can be directly matched to the electron density determined by the LOPP Raman at the highest excitation point, and thus, the needed scaling parameter *W*_r0_ ≈ 3.3 × 10^4^ s^−1^ is obtained for use in Eq. 5. This parameter is the radiative recombination rate associated with the background doping. Then, the *n* vs. *P* curve is obtained over the full range of excitation densities, as shown in Fig. 4a. In the excitation density region where Raman results are available, the densities agree quite well. We can further calculate *W*_r_ vs. *P* for the sample, as shown in Fig. 3b, that exhibits a significant excitation density or carrier density dependence, similar to the reported carrier density dependence of the radiative decay time with varying doping level^30^. For instance, for WA540, it varies from *W*_r_ = 3.5 × 10^7^ s^−1^ at 0.5 W cm^−2^ to 7.6 × 10^8^ s^−1^ at 5 × 10^3^ W cm^−2^.

Assuming there is only one dominant type of nonradiative recombination center, and utilizing the charge neutrality condition, *δp* = *n* + *fN*_t_ (with *δp* = *p*_0_*dP*^*ζ*^), we can further obtain *p*_0_ and *N*_t_ by fitting two sets of data (*P*, *f*) and (*P*, *n*) simultaneously, which yields *p*_0_ ≈ 2.2 × 10^13^ cm^−3^ and *N*_t_ ≈ 1.7 × 10^17^ cm^−3^. Once *p*_0_ and *N*_t_ are known, essentially all recombination parameters can be derived and the corresponding rates can be evaluated at any excitation density: the inter-band radiative recombination coefficient *B* = *W*_r0_/*p*_0_ ≈ 1.5 × 10^−9^ cm^3^ s^−1^; the defect state recombination coefficient *B*_t_ = *W*_t0_/*p*_0_ ≈ 2.7 × 10^−10^ cm^3^ s^−1^ where *W*_t0_ (= *β*_0_/*N*_t_) ≈ 5.9 × 10^3^ s^−1^ is the defect state recombination rate determined by the background doping, and thus, the maximum majority carrier (hole) capture rate *c*_M_ = *B*_t_*N*_t_ ≈ 4.6 × 10^7^ s^−1^; the minority carrier (electron) capture rate *c*_t_ = *W*_r0_/*μ*_0_ ≈ 9.0 × 10^10^ s^−1^ or coefficient *γ*_t_ = *c*_t_/*N*_t_ ≈ 5.2 × 10^−7^ cm^3^ s^−1^ or cross-section *σ*_t_ = *γ*_t_/*v*_th,e_ ≈ 8.7 × 10^−15^ cm^2^ (*v*_th,e_ = 6 × 10^7^ cm/s is the thermal velocity for electrons in GaAs at 300 K); the majority carrier (hole) capture cross-section *σ*_tM_ = *B*_t_/*v*_th,h_ ≈ 1.5 × 10^−17^ cm^2^ (*v*_th,h_ = 1.8 × 10^7^ cm/s is the thermal velocity for holes in GaAs at 300 K); the majority carrier density *p* = *p*_0_(1 + *dP*^*ζ*^) as shown in Fig. 4a; the minority carrier (electron) nonradiative recombination or capture rate (equivalent to the *A* coefficient of the ABC model) *W*_nr_ = *c*_t_(1 − *f*), along with the total minority carrier recombination rate *W* = *W*_r_ + *W*_nr_, as shown in Fig. 3c; and the majority carrier (hole) nonradiative recombination rate or capture rate *R*_t_ = *B*_t_*N*_t_*f*, which describes the rate of the minority carriers depleted from the defect state by recombing with a free hole, along with the total majority carrier recombination rate *R* = *Bn* + *R*_t_, as shown in Fig. 3d. Clearly, the total recombination rates, *W* and *R*, are both dominated by nonradiative recombination in the low to middle level excitation region. The derived recombination-related parameters are summarized in Table 1.

For an n-type sample, such as 1-1138 and B2206, the Raman results yield the majority carrier densities. By matching to the electron density determined by the LOPP Raman at the highest excitation level, we can obtain the background doping level *n*_0_, and thus, *n* vs. *P* for the whole range of the excitation densities. The results for 1-1138 and B2206 are shown in Fig. 4b, c, respectively, which show fairly good agreement with the Raman results. Similarly, by applying the charge neutrality condition, *δn* = *W*_r0_*p*_rel_ + *fN*_t_, where *δn* = *n*_0_*dP*^*ζ*^ has been determined above, *p*_rel_ is the relative minority carrier density *p* evaluated with Eq. 5 without *W*_r0_, we can obtain *W*_r0_ and *N*_t_ by fitting two sets of data (*P*, *f*) and (*P*, *p*_rel_) simultaneously. *W*_r_ vs. *P* can then be calculated, as shown in Fig. 3b for 1-1138 and B2206. Thereafter, the other two recombination rates, *W*_nr_ and *R*_t_, can be calculated, as shown in Fig. 3c, d, respectively; and all of the remaining recombination parameters can be derived. The results for 1-1138 and B2206 are also summarized in Table 1. The step-by-step procedures for carrying out the above analyses are summarized in diagrams for p- and n-type material, respectively, in Note 2 in SI.

For comparison, we also evaluate the carrier densities using the PL and Raman data with the 100× lens as shown in Fig. 4d–f using the results in Fig. S2c. For WA540 in Fig. 4d, at high excitation density, the minority carrier density agreement between the Raman and PL results is fairly good, which may be explained by the relative dominance of radiative recombination over diffusion loss at high excitation density. However, going into the lower excitation region, the Raman result becomes higher, perhaps because the Raman measurement is not precise enough to probe the small change in carrier density. For 1-1138 and B2206, the majority carrier densities probed by Raman and PL agree reasonably well in the very high density region, but the densities obtained by Raman become progressively lower than those obtained by PL with decreasing excitation because the two probe techniques have different sensitivities to the diffusion effect. The intent of this brief discussion on the 100× results is to point out that the applicability of the approach is limited by carrier diffusion. To take advantage of the high spatial resolution of the diffraction-limited beam size, lateral diffusion should be explicitly considered, which would be an extension of the current work.

## Discussion

The *I*_PL_ vs. *P* dependence of WA540 in Fig. 2b, a stretched S shape, represents the characteristic of a material with a moderate level of defect and background doping density, where defects dominate the low excitation density region, they are being saturated in the transition region, and the high excitation density region is dominated by bimolecular radiative recombination. Such a functional form resembles the *I*–*V* characteristic of a device in a space charge limited current mode, which is determined by the interplay of background free charges, trap states, and externally injected carriers^31^. The excellent fitting for WA540 seems to suggest that the single-defect-center approximation works well because the energy profile of the defect states can significantly affect the shape of the functional form of the transition region^31^. 1-1138 could be viewed as a more extreme version of WA540, where radiative recombination below the transition is too weak to be detectable. The fitting yields a much higher minority carrier capture rate *c*_t_ ≈ 4.2 × 10^12^ for 1-1138 vs. 9.0 ×10^10^ s^−1^ for WA540. The lower quality fitting might also suggest that the single defect center approximation is inadequate. On the other hand, B2206 represents a distinctly different characteristic of nonradiative recombination, namely, having a much smaller *c*_t_ ≈ 6.3 × 10^8^ s^−1^, because of a much smaller *γ*_t_, despite of a high *N*_t_ ≈ 1.0 × 10^19^ cm^−3^ (compared to 1.7 × 10^17^ and 1.3 × 10^16^ cm^−3^ for WA540 and 1-1138), and a larger majority capture cross-section *σ*_tM_ ≈ 1.8 × 10^−16^ cm^2^ (compared to 1.5 × 10^−17^ and 7.6 × 10^−17^ cm^2^ for WA540 and 1-1138). These differences allow B2206 to have significantly higher PL efficiencies in the low excitation density region due to the smaller *c*_t_, but the defect centers are less likely to be saturated due to the larger *N*_t_ and *σ*_tM_, resulting in much smaller *f* values as shown in Fig. 3a. Thus, B2206 has lower PL efficiencies in the high excitation region, which yields an overall smoother excitation density dependence compared to WA540.

The derived minority and majority carrier cross-sections are generally in the range of previously reported values for GaAs^2,32^. The derived values for the inter-band recombination coefficient B, 1.5 × 10^−9^, 1.6 × 10^−9^, and 2.7 × 10^−9^ cm^3^/s, respectively, for WA540, 1-1138 and B2206, are of comparable order with the literature values of 10^−10^ to 10^−9^ cm^3^/s^33–35^. The values for the defect recombination coefficient *B*_t_, 2.7 × 10^−10^, 4.6 × 10^−9^, and 1.1 × 10^−8^ cm^3^/s, respectively, for WA540, 1-1138 and B2206, seem to be reasonable in magnitude. If the defect recombination is radiative, a smaller value than *B* is expected, because the defect state tends to be a mixture of bulk-like states of different symmetries. If the defect recombination is nonradiative (e.g., through phonon emission), *B*_t_ can be either larger or smaller than *B*.

It is worth commenting on the connection between our coupling model and the SRH model. In the latter, the recombination rate through the defect state is given below^12^:

6 $$U = \frac{{\left( {np - n_{{{\mathrm{i}}}}^2} \right)}}{{C_{{{\mathrm{p}}}}^{ - 1}\left( {n + n_1} \right) + C_{{{\mathrm{n}}}}^{ - 1}\left( {p + p_1} \right)}}$$

where *C*_n_ is the electron capture rate, *C*_p_ is the hole capture rate, and *n*_1_ and *p*_1_ are terms related to electron and hole emission to the respective band edge. In this model, the inter-band radiative recombination is not explicitly considered, but may be implicitly reflected in *n* and *p*. The coupling model is equivalent to the SRH model under the condition of *n*_1_ ≈ *p*_1_ ≈ 0, which also implies *n*_i_ ≈ 0, but explicitly includes radiative recombination. The approximate result of Eq. 6 can be readily obtained from Eq. S2. For instance, for a p-type semiconductor, *C*_n_ is equivalent to *c*_t_ = *γ*_t_*N*_t_, the minority carrier capture rate at the low excitation limit, whereas *C*_p_ is equivalent to *c*_M_ = *B*_t_*N*_t_, the majority carrier capture rate at the high excitation limit. When plotted, *U* is exactly identical to *c*_t_(1 − *f)n* = *c*_M_*fp*. The limitations of the ABC model can be more clearly explained by Eq. 6 and our results. For a p-type semiconductor, the A term of the ABC model can be obtained by further assuming $C_{\rm{n}}^{ - 1}p \gg C_{\rm{p}}^{ - 1}n$ or *C*_p_*p* ≫ *C*_n_*n*, then *A* = *C*_n_. This condition implies that at the defect state, the depletion of the captured electrons to the valance band is much faster than being captured from the conduction band such that *f* ≈ 0 can be maintained. This assumption decouples the free carrier rate equation from that of the defect state, which also makes it impossible to obtain the detailed information about the defect from PL. This assumption is apparently invalid in the general case. For instance, for WA540, at high excitation density, *n* ∼ *p*, instead of *C*_p_ ≫ *C*_n_, the opposite is true, i.e., *C*_p_ (*c*_M_) ≪ *C*_n_ (*c*_t_), according to our analysis (Table 1).

The ABC model further assumes *n* = *p* for any excitation density, which is apparently invalid for the low excitation density region, as shown in Fig. 4. A comparison is offered, as Fig. S4, for WA540 for the *n* vs. *P* and *p* vs. *P* curves derived from the coupling model and the single *n* vs *P* curve from the ABC model.

Compared with a typical DLTS measurement, the current approach is able to yield as much information about the defect state, if not more, because conventional reverse-bias DLTS usually measures only the majority carrier capture cross-section and defect density. DLTS studies of minority carrier trap properties typically require forward bias pulses or optical excitation, which introduces ambiguity in trap identification^36^. However, DLTS can also provide information regarding the energetic position of the defect state, while the current version of our optical approach cannot. Since DLTS obtains trap depth by measuring the temperature-dependent carrier emission rate, in principle, our optical method could employ changing temperature to obtain comparable information, although with increased complexity in data analysis. A potential limitation of the current optical approach is the assumption of a single defect state when the charge neutrality condition is applied, which may not be realistic for the whole range of excitation densities under investigation. It has been pointed out that the spectrum of the defect states may affect the shape of the transition region^37,38^. In reality, we have found that the fitting quality in the step to extract *N*_t_ is adequate only for the high excitation density region, which implies that the empirical power-dependent radiative recombination rate might reflect the contributions from more than one defect state, although there might be a dominant one.

As an approach to investigate the nonradiative recombination process, the coupling model is intended for moderate- to wide-bandgap semiconductors and the low to moderate excitation region before the Auger effect becomes significant. The applicability of the approach to a specific sample is mostly determined by the ability to get a good fit of the *I*_PL_ vs. *P* curve using Eq. 3, which has been demonstrated for a variety of halide perovskite, CdTe, and GaAs samples^5^. The Raman result is primarily needed to determine a scaling constant, which yields the background doping level. If the Raman data is not obtainable, one could attempt to obtain the background doping through other means, such as a Hall measurement. Even though the methodology has only been used to analyze PL results, it is expected to be applicable to EL as well.

## Summary and conclusion

A simple all-optical approach has been shown to be capable of revealing surprisingly more information than one might normally expect about carrier recombination processes. Particularly, when combined with the LO-phonon/electron-plasmon coupled mode Raman measurement, practically all radiative and nonradiative recombination parameters, including related efficiencies, occupation states, rates, coefficients, densities, and cross-sections, can be determined quantitatively with few approximations. This study is the first demonstration of an all optical technique that can yield comprehensive and quantitative information about defect states, including density and cross-sections, which has historically only been obtainable via electrical techniques like DLTS. A practical procedure, which is slightly different for n- and p-type material, is described for systematically analyzing the PL and Raman data. Prototype samples of GaAs double-heterostructures exhibit two distinctly different functional forms of PL intensity vs. excitation density, representing two very different nonradiative recombination characteristics. Our ability to explain these divergent responses suggests the general applicability of this approach and the potential to further develop into a complementary defect characterization tool, deep level optical spectroscopy (DLOS).

## Materials and methods

Three (001) GaAs double heterojunction (DH) samples are used in this study. In all structures, both interfaces are passivated to minimize surface recombination. The first is a 2 μm GaAs layer sandwiched between two 50 nm GaInP layers, which are all nominally undoped with an estimated n-type background doping level of ~5 × 10^14^ cm^−3^ (“1-1138”). The second has a 0.5 μm GaAs layer with a 0.1 μm lower barrier and 5 nm upper barrier of GaInP and an estimated p-type background doping level of ~10^15^ cm^−3^ (“WA540”). Both were grown by MOCVD but in different systems. The third is Al_0.4_Ga_0.6_As/GaAs/Al_0.4_Ga_0.6_As with 1 μm of GaAs and 30 nm Al_0.4_Ga_0.6_As barriers grown by MBE with an n-type background doping level in the mid-10^15^ cm^−3^ (“B2206”). Details of the growth methods can be found elsewhere^6,39,40^.

The measurements were conducted with a Horiba LabRAM HR800 confocal Raman microscope with a 1200 g/mm grating. A 532 nm laser was used as the excitation source in backscattering configuration. For all Raman spectra, a 650 nm short pass filter was placed in front of the detector to remove stray light from GaAs photoluminescence. The carrier diffusion lengths of these GaAs DH samples were measured previously to be ~5 μm under moderately high to high excitation densities^5,41^. To examine the effect of carrier diffusion, data were taken using 10× (NA = 0.25) and 100× (NA = 0.9) microscope lenses. The excitation density was estimated as *P* = laser power/A, where A is the area determined by the full width at half maximum of the measured laser profile. The spot sizes were 6.0 and 0.72 μm, respectively, for the 10 × and 100× lenses. The laser power was measured at the exit of the microscope lens. The laser power (16 mW of full power) was changed either using built-in attenuators D1–D4, giving approximately 1–4 orders of attenuation, or by reducing the operational current of the laser. The power density varies from below 0.1 W/cm^2^ (about terrestrial 1 sun) to around 10^5^ W/cm^2^. All measurements were performed at room temperature under ambient conditions. To obtain the actual laser power absorbed by the sample, surface reflection loss R has been removed by multiplying the laser power by the factor (1−*R*). *R* was measured to be 0.37 at 532 nm for GaAs. We have examined the possible laser-induced heating effect using the anti-Stokes/Stokes Raman peak intensity ratio^42^ and confirmed that up to the highest power used the heating effect is minimal (8 K) for this experiment.

## Supplementary information

Supplementary Information

## Data Availability

The data that support all plots within this paper is available from the corresponding author upon reasonable request.
