# Supplementary material for: An all optical approach for comprehensive in-operando analysis of radiative and nonradiative recombination processes in GaAs double heterostructures
Source: Light Sci Appl. 2022 May 13;11:137. doi: 10.1038/s41377-022-00833-5 (PMC9106719; doi:10.1038/s41377-022-00833-5)
Supplement: Supplementary file 1 — Supplementary Information [file 41377_2022_833_MOESM1_ESM.docx]

**Supplementary Information for**

**An all optical approach for comprehensive in-operando analysis of radiative and nonradiative recombination processes in GaAs double heterostructures**

Fan Zhang^1,2^, Jose F. Castaneda^1^, Timothy H. Gfroerer^3^, Daniel Friedman^4^, Yong-Hang Zhang^5^, Mark W. Wanlass^6^, and Yong Zhang^1,*^

^1^ Department of Electrical and Computer Engineering, The University of North Carolina at Charlotte, Charlotte, NC 28223, USA

^2^ Songshan Lake Materials Laboratory, Dongguan, Guangdong 523808, China

^3^ Department of Physics, Davidson College, Davidson, North Carolina 28035, USA

^4^ National Renewable Energy Laboratory, Golden, CO 80401, USA

^5^ School of Electrical, Computer and Energy Engineering, Arizona State University, Tempe, AZ 85287, USA

^6^ Wanlass Consulting, Norwood, CO 81423, USA

* [yong.zhang@uncc.edu](mailto:yong.zhang@uncc.edu)

This document contains Supplementary Note 1, 2 and Supplementary Figures S1 – S4.

**Supplementary Notes**

**Note 1: Coupling Model**

**
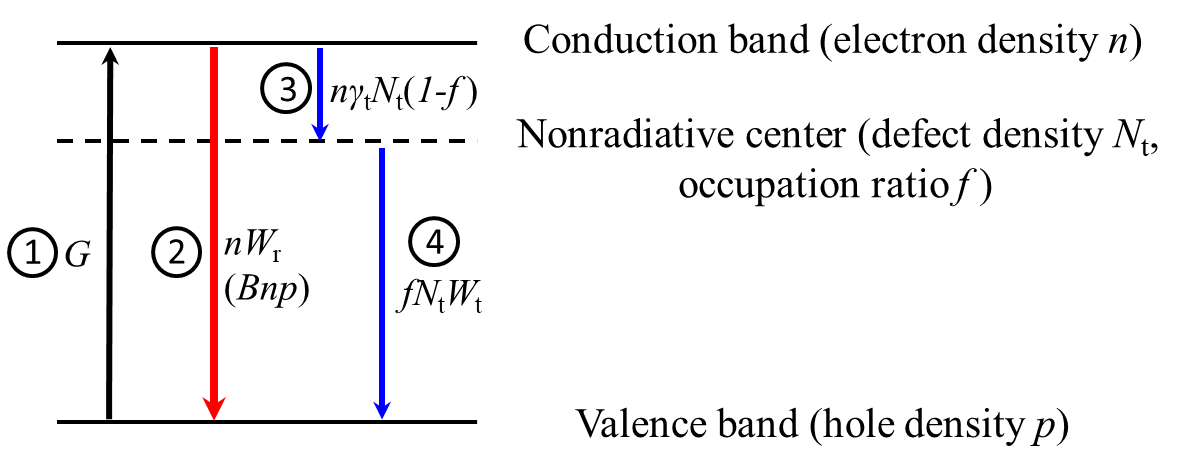
**

$\frac{dn}{dt}=G-nW_{r}-nc_{t}\left( 1-f \right)+fN_{t}e_{t}=0$ (S1)

$\frac{dN}{dt}=nc_{t}\left( 1-f \right)+e_{p}N_{t}\left( 1-f \right)-fN_{t}W_{t}-fN_{t}e_{t}=0$ (S2)

In the band diagram, arrows 1-4 describe different processes of photo-excited electrons in a *p*-type semiconductor. 1: Generation; 2: band edge emission; 3: defect capture; 4: defect recombination. Nonradiative recombination consists of two steps: process 3 and 4. The corresponding rate equations are given by Eqs. (S1) and (S2). Thermal excitation of electrons from the defect state to the conduction band, the *e*_t_ term, and from the valance band to the defect state, the *e*_p_ term, is assumed negligible. Defect occupation ratio *f* (=*N*/*N*_t_, *N* is the electron density at the deep defect state) is excitation level dependent, which describes the main difference between the coupling model and ABC model. Eqs. (S1) and (S2) are the steady-state rate equations for electron density *n* in the conduction band and electron occupation fraction *f* at the defect state. All other parameters in the two equations are defined in the main text below Eq. (3). The simultaneous solutions of (*n*, *f* ) of Eqs. (S1) and (S2) yield Eqs. (3) and (4) in the main text.

If the semiconductor is *n*-type, holes will be the minority carriers and we only need to change *n* to *p* in Eq. (3) and (4).

**Note 2: Fitting Procedure Diagram**

**
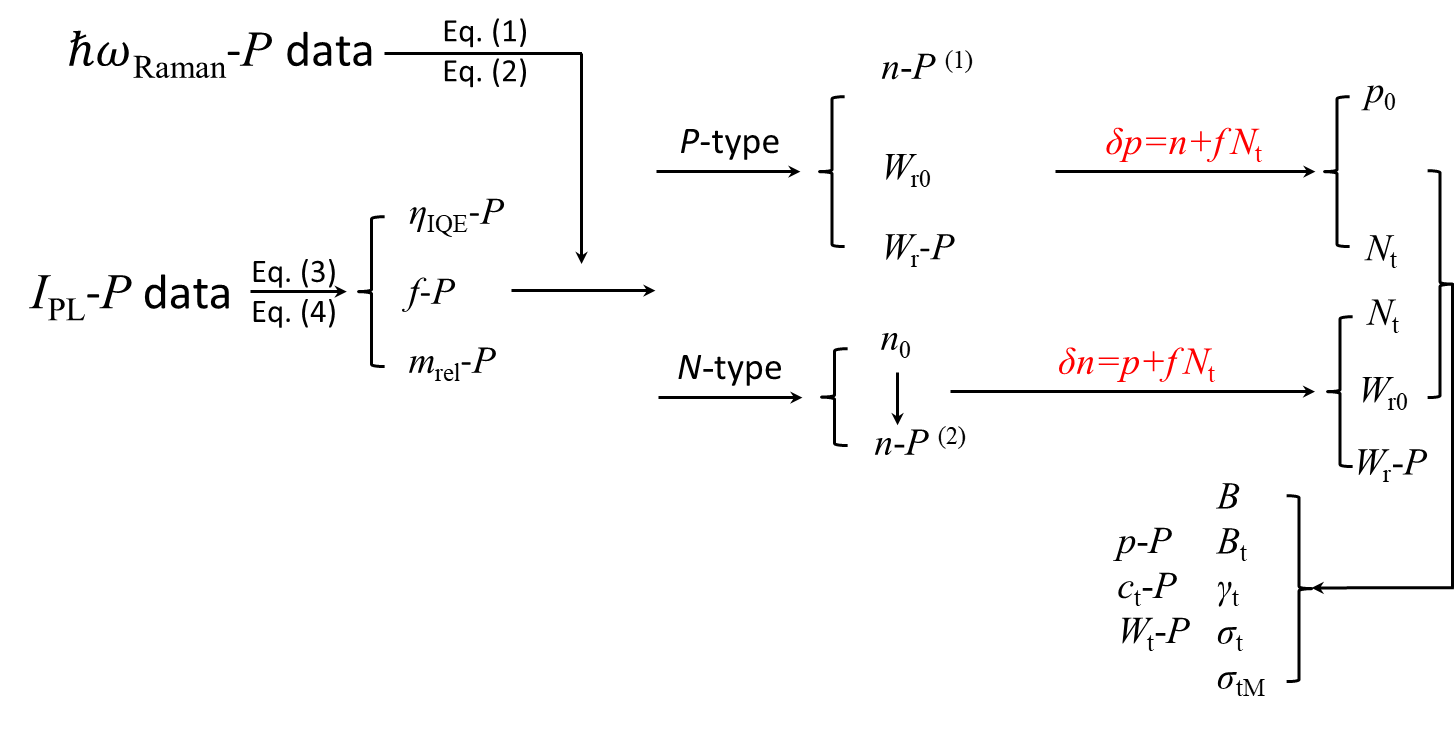
**

*m*_rel_: relative value of the minority carrier density, *n* for *P*-type and *p* for *N*-type, thus, *W*_r_=*Bp* for *P*-type and *W*_r_=*Bn* for *N*-type; the definition of other symbols can be found in Table 1.

(1) & (2): We get *n*-*P* (electron density-power density) relationship by aligning LOPP data (electron density *n*) to relative minority carrier *m*_rel_ at high power density region, then minority carrier radiative recombination rate *W*_r_ (*P*-type) and background doping level *n*_0_ (*N*-type) can be derived. With the help of charge neutrality condition, all parameters listed can be obtained. Under the mediate to high excitation density, the photoexcited carrier density is much higher than the background doping level and minority carrier density.

**Supplementary Figures**

**Figure S1**

**Figure S1. PL spectra of three GaAs DH samples under 10× lens at different power densities. Different noise levels are due to acquisition parameters (integration time and accumulation). However, they have minor influences on PL intensity in unit of counts/second.**

The PL lineshapes of all GaAs samples in main text show minimal changes when changing the excitation density (Figure S1). The constant peak position and slope on the short wavelength side suggest that carriers in the band are well thermalized. As a result, we collect only one spectral window near 870 nm to get the PL intensities in Figure 2b in the main text.

**Figure S2**

**Figure S2. Excitation power dependent PL data for all three samples (with 100× lens). (a) PL intensity vs. power density (*P*); (b) PL/*P* vs. *P*; and (c) IQE vs. *P*. Solid symbols are experimental data; solid lines are fitting curves with the coupling model.**

**Figure S3**

**Figure S3. Excitation power density dependent IQE data (with 10× lens) for all three GaAs samples fitted by the ABC model. Solid symbols are the experimental data of Figure 2c scaled by the fitting parameter k; solid lines are the fitting curves.**

We attempted to fit data in Fig. 2b with the ABC model: $\eta_{EQE}$= *k*/(1 + *a*/√*I*_PL_ + *c*√*I*_PL_), where *k* is a scaling parameter, *a* and *c* are the corresponding parameters related to the A and C term, respectively. IQE can be obtained through *η_IQE_* = *η_EQE_*/*k*. The fitting results are shown in Fig. S3. The maximum efficiency is 19.3% for WA540, 36.6% for 1-1138 and 66.4% for B2206. Despite the fitting results seem to be rather good in the high power region, but much worse in the full range than the coupling model fitting results shown in Figure 2d. Furthermore, the relative efficiency between the WA540 and B2206 in the high power region is opposite to the experimental data, where WA540 was found to be significantly more efficient than B2206 (by about 33%).

**Figure S4**

**Figure S4. Comparison between the coupling and ABC model derived photo-excited carrier densities for WA540 under 10× lens.**

In the ABC model, carrier density *n* (= *p*) can be obtained through *n* =$\sqrt{IQE\cdot G/B}$. Using the IQE result given in Fig. S3, the carrier density is calculated for WA540 for the purpose of illustrating the contrast between the two approaches. In the coupling model, *n* and *p* are very different in most part of the excitation density range studied, whereas in the ABC model, *n* = *p* is assumed, thus, the single curve is found to be in between the two. Note that the ABC model itself cannot yield the carrier density without taking the *B* value from the literature where the *B* value varies significantly. In contrast, the *B* value in our approach can be obtained self-consistently. We have used the *B* value from our analysis in the ABC model.
